# Pseudocholinesterase Deficiency in Ambulatory Surgery: A Case Report

**DOI:** 10.7759/cureus.80492

**Published:** 2025-03-12

**Authors:** Elizabeth Johnson-Gray, Austin J Shaffer, Priyanka Pandey, Greeshma Allareddy, Matthias Franzen

**Affiliations:** 1 Anesthesiology, OhioHealth Doctors Hospital, Columbus, USA; 2 Anesthesiology, Cleveland Clinic, Cleveland, USA

**Keywords:** dibucaine, pseudocholinesterase (pche), pseudocholinesterase (pche) deficiency, succinylcholine, train-of-four monitoring

## Abstract

Pseudocholinesterase (PCHE) deficiency is a rare condition that results in increased sensitivity to certain medications, including the paralytic agents succinylcholine and mivacurium. PCHE deficiency typically presents when patients cannot be weaned off the ventilator because of residual paralysis. These patients require mechanical ventilation and sedation until they are strong enough to be safely extubated. This report presents the case of a 53-year-old male patient whose only known surgical and anesthetic complication was post-op nausea and vomiting and who was diagnosed with PCHE deficiency after he could not be safely extubated in the operating room (OR) after a robotic inguinal hernia repair. The patient was admitted to the ICU and successfully extubated several hours later.

## Introduction

Pseudocholinesterase (PCHE) is a glycoprotein enzyme also known as butyrylcholinesterase; its function is to break down, via hydrolysis, exogenous-based choline esters, such as succinylcholine and mivacurium, and ester local anesthetics, such as cocaine [1]. PCHE is produced in the liver, and the breakdown process occurs before these esters reach the neuromuscular junction [2]. This enzyme is found in many human tissues but not in red blood cells. PCHE deficiencies are rare, occurring in one in every 3200-5000 patients. The deficiency tends to be more prevalent in Caucasian males of European descent, the Persian Jewish community, and Alaska natives [1].

A patient who is heterozygous for PCHE will exhibit a prolonged response to the depolarizing neuromuscular blocker succinylcholine [3,4]. Patients who are homozygous atypical exhibit a marked prolonged response to succinylcholine, anywhere from four to eight hours [2]. PCHE deficiency has no preceding signs or symptoms until it presents itself as delayed emergence from anesthesia due to the body’s limited ability to break down the exogenous-based choline esters introduced during the anesthetic induction process. The patient will not be able to be weaned off the ventilator and will have insufficient twitches.

The mainstay treatment for this condition is to monitor the patient, aid their respiration, and maintain sedation until they regain neuromuscular function [4]. Diagnosis of this condition is done by testing the PCHE level and dibucaine number. Decreased PCHE and a dibucaine number less than 30 correlate with an increased risk of prolonged paralysis [5].

## Case presentation

A 53-year-old male Caucasian patient, 107 kg (BMI of 34.5), presented for elective robotic right inguinal hernia repair, American Society of Anesthesiologists (ASA) 2. The patient’s medical history included gastroesophageal reflux disease, migraines, and tobacco use disorder. The patient had an anesthetic history of post-op nausea and vomiting. His surgical history included bilateral inguinal hernia repair, foot fracture surgery, and forearm fracture surgery. His family history noted hypertension, migraines, and brain cancer in his mother. The only allergy the patient endorsed was a penicillin allergy. In the preoperative evaluation, the patient was afebrile (36.6°C) and normotensive at 128/78, with SPO_2_ at 97 and respiratory rate at 14. CBC and CMP were unremarkable except for mild elevation of his glucose at 136. 

On induction of general anesthesia, the patient received 2 mg IV of midazolam, 50 mcg IV of fentanyl, 100 mg of lidocaine IV, 200 mg of propofol IV, 10mg of rocuronium (as a defasciculating dose), and 100 mg succinylcholine The patient was intubated with an 8 mm cuffed oral endotracheal tube, with an initial ETCO_2_ of 33 mmHg. The patient was then given 20 mg of rocuronium. General anesthesia was maintained with sevoflurane, with the patient maintaining normothermia during the case. 

Toward the end of the procedure, tetralogy of Fallot (TOF) was assessed and found to be 1/4 twitches. The patient was reversed with 400 mg of sugammadex. Pressure support ventilation was attempted; however, the patient was placed back on synchronized intermittent mandatory ventilation (SIMV) because of insufficient tidal volumes. A few minutes later, the TOF was reassessed, and the patient still had one twitch. TOF was assessed on the adductor pollicis and confirmed to be 1/4 twitches. Another 400 mg of sugammadex was given. 

The patient was still unable to tolerate PSV and was pulling tidal volumes of less than 200 on SIMV. TOF at this time was still 1/4. The patient was able to lightly squeeze his hands and lightly lift his thumb to answer yes or no questions. Flumazenil was given to rule out potential overdose. A total of 0.5 mg was given, with no improvement in respiratory status. 

The patient was continued on SIMV, and considerations to other causes of prolonged blockade were considered. The patients’ pupils were assessed to rule out the possibility of a cerebral vascular accident. The patient’s scopolamine patch was removed. Complete blood count (CBC), comprehensive metabolic panel (CMP), magnesium, PCHE level, and point-of-care glucose were drawn to evaluate potential causes. The dibucaine number was not drawn because the test is not available at our institution. The patient was normothermic during the entirety of the case, so prolongation due to hypothermia was ruled out. The decision was made not to extubate and to instead inform the ICU and transfer to PACU intubated. The decision was discussed with the patient, who was able to lightly squeeze his hand and move his thumb up and down at the time. The patient’s wife was informed, and on transport to the PACU, the patient was given dexmedetomidine and propofol pushes. The patient was started on a dexmedetomidine drip of 0.5 mcg/kg per hour in the PACU. 

While the patient was in the PACU, the CBC returned with no abnormalities. CMP showed mild hyponatremia at 134 and mild hypocalcemia at 8.2. Point-of-care glucose was 114. Magnesium was normal at 1.9. The lab called and informed the team that it would take several days for the PCHE level to come back. Four hours after succinylcholine administration, TOF had improved to 2/4, and the patient was able to slightly nod his head yes and no and use his index finger to write letters on the staff’s hands. Pressure control ventilation was tried again, but he requested to be placed back on the ventilator after a few minutes because he was struggling to breathe. 

The patient was transferred to the ICU on a dexmedetomidine drip and was successfully extubated seven hours after succinylcholine administration. The patient was discharged home on POD 1 with full clearance from physical therapy. His PCHE levels returned to POD 3 at 2,565, with normal levels ranging from 5,320 to 12,920. The patient was called and informed of his results and counseled on his condition and the importance of further testing with dibucaine number. He was informed that his condition can be inherited, and it was recommended that his children be tested. The patient most likely was homogenous atypical. He was unable to be extubated until more than seven hours after the administration of succinylcholine. 

On POD 12, the patient presented to the general surgeon’s office for a postoperative follow-up. He denied any complaints at his follow-up and stated that he was feeling well. At the time of this publication, no dibucaine number testing has been documented. 

## Discussion

PCHE deficiency is an enzymatic deficiency in the PCHE gene, located on chromosome 3; a consequence is that the body cannot adequately break down certain drugs, such as succinylcholine [3]. This deficiency is inherited in an autosomal recessive pattern, meaning that both copies in each cell must have mutations. This deficiency occurs in one in every 3200-5000 people. It tends to be more prevalent in Caucasian males of European descent, the Persian Jewish community, and Alaska natives [1].

PCHE deficiency can also have nongenetic causes or be acquired. Kidney or liver disease, malnutrition, pregnancy, and major burns are some of the causes of acquired PCHE deficiencies. When acquired deficiency occurs, this condition is not inherited and cannot be passed to the next generation [2]. 

This condition is typically found incidentally when a patient cannot be weaned from the ventilator after the administration of succinylcholine. Typically, succinylcholine is broken down within minutes of being administered. A patient who is heterozygous or homozygous for the deficiency can exhibit a different response. Patients who are heterozygous tend to have a shorter period of prolonged muscular blockade, anywhere from 30 to 60 minutes. By contrast, patients with homozygous deficiency have a prolonged muscular blockade of four to eight hours [1,2].

This deficiency is often diagnosed after the patient has been given one of the drugs listed above and has exhibited a prolonged blockade. In patients who experience prolonged blockade, it is important to rule out other causes of the blockade [6,7]. Electrolyte imbalances, hypoglycemia, liver disease, renal disease, and drugs given intraoperatively, such as furosemide and magnesium that inhibit acetylcholine formation and release, can impact the neuromuscular blockade. 

In our patient, basic electrolytes, labs, and point-of-care glucose were all normal. The PCHE level was obtained to help diagnose his condition. The dibucaine number is typically the diagnostic of choice to diagnose this condition because dibucaine is a local anesthetic that will inhibit the activity of the normal variant of PCHE enzyme by 80%. When dibucaine is exposed to the activity of atypical variants of the PCHE enzyme, the activity is reduced to a much smaller degree. A normal dibucaine number falls between 70 and 80, but a heterozygous enzyme deficiency falls between 50 and 60, and a homozygous enzyme deficiency falls between 20 and 30 [4] (Table 1).

**Table 1:** Type of pseudocholinesterase

Incidence	Dibucaine number	Response to succinylcholine	Type of pseudocholinesterase
Normal	70–80	Normal	Homozygous (normal)
1/480	50–60	Lengthened 50%	Heterozygous atypical
1/3200	20–30	Prolonged four to eight hours	Homozygous atypical

In the case of our patient, our institution did not offer a test for dibucaine number, so the patient was encouraged to get further testing in the upcoming months. He was informed of the likelihood that he is homozygous for this deficiency, and he was counseled on the importance of telling his future anesthesia providers about his likely condition. It was also recommended for the patients’ family members to undergo testing to determine whether they had this condition.

Mechanical ventilation and sedation are the mainstays of treatment until the respiratory muscle paralysis resolves. Recovery takes place when succinylcholine passively diffuses away from the neuromuscular junction. It is vital for the patient to be adequately sedated until symptoms resolve [5]. Our patient was sedated with a dexmedetomidine infusion; however, other case reports have used propofol.

As mentioned above, once the patient has been successfully extubated, it is important to discuss the new diagnosis with the patient and their family and advise them on further laboratory testing and further testing for their family members. Patients with PCHE deficiency should be counseled on their condition and the importance of telling their future anesthesia providers about it. 

## Conclusions

This case illustrates the diagnosis and management of a patient suspected of PCHE deficiency. These patients should not be extubated until they can protect their airways, have a strong cough, and are capable of following commands. In addition, consideration should be given to ensure that these patients are adequately sedated until normal neuromuscular function is returned. ​​In addition, these patients should be counseled on the possibility of other family members having this deficiency as it can be inherited and to inform their future anesthesia providers. Patients with this deficiency should not receive succinylcholine, and drugs like mivacurium should be used with caution.
